# From Mistrust to Malice: Examining the Influence of Adverse Childhood Experiences on Reactive and Appetitive Aggression in Male Forensic Psychiatric Patients with a History of Drug Abuse Through the Lens of Psychodynamic Personality Structures

**DOI:** 10.3390/bs15030246

**Published:** 2025-02-21

**Authors:** Michael Fritz, Sylvia Flad, Judith Streb, Manuela Dudeck

**Affiliations:** Department of Forensic Psychiatry and Psychotherapy, Medical Faculty, Ulm University, 89312 Günzburg, Germany

**Keywords:** operationalized psychodynamic diagnosis, appetitive aggression, reactive aggression, adverse childhood experiences

## Abstract

Adverse childhood experiences (ACEs) represent one of the most critical factors contributing to the manifestation of psychiatric disorders later in life. Furthermore, such experiences are often associated with deficits in interpersonal relationships, manifesting as mistrust and violent behaviors, and are indicative of a fragmented personality. This study aimed to analyze the correlative relationships between personality deficits influenced by ACEs and the expression of reactive and appetitive aggression using self-report questionnaires in 53 male forensic psychiatric patients with a drug dependency background detained under §64 of the German Criminal Code between 2019 and 2022. Instruments included the Operationalized Psychodynamic Diagnosis Structure Questionnaire (OPD-SF), the Maltreatment and Abuse Chronology of Exposure Scale–German Version (KERF), and the Appetitive and Facilitative Aggression Scale (AFAS). Specifically, the OPD-SF used the following subscales: self-perception, self-regulation, the regulation of object relations, emotional communication inward/outward, internal/external attachment, and total score. The results demonstrate a significant relationship between childhood traumatic experiences, personality structure, attachment capacity, self-perception, and regulation and the expression of both reactive and appetitive aggression. While the association with reactive aggression is intuitively plausible, the findings notably reveal that the propensity to derive pleasure from violence is also associated with personality deficits caused by adverse childhood experiences. These findings have important implications for the treatment of offenders with personality disorders and should be considered in therapeutic interventions.

## 1. Introduction

Adverse childhood experiences (ACE) encompass a broad range of stressful or traumatic events occurring before the age of 18, such as physical, emotional, and sexual abuse, as well as emotional and physical neglect (Gilgoff et al., 2020). Expectedly, research has extensively documented the significant impact of ACEs on psychosocial and physiological development. Physiologically, ACEs have been associated with chronic conditions, such as obesity and diabetes, while their psychological consequences include heightened risks for post-traumatic stress disorder (PTSD), emotionally unstable personality disorders, depression, and aggression (Almeida et al., 2024; Herzog & Schmahl, 2018).

Despite these insights, the relationship between ACEs and subtypes of aggression (reactive, instrumental, and appetitive (Fritz et al., 2023)) remains currently (especially in the context of appetitive aggression) insufficiently understood and warrants further investigation. Notably, a recent study underlined the impact of ACEs, particularly childhood maltreatment, on heightened aggression in a different forensic psychiatric patient population (Koolschijn et al., 2023). In this study, emotional, physical, and sexual abuse, as well as physical neglect, were associated with both reactive and instrumental aggression. Furthermore, emotional neglect specifically contributed to increased instrumental aggression.

Traditional models of human aggression distinguish exclusively between these two fundamentally different forms of aggression. Here, reactive aggression occurs as a response to an acute stimulus, perceived as threatening, while instrumental aggression is goal-oriented and self-motivated and involves harmful behavior aimed at obtaining positive reinforcers (Vitiello & Stoff, 1997). The third type—appetitive aggression—is characterized by the pleasure and satisfaction derived from acts of violence. To date, it has primarily been conceptualized in the context of extreme trauma, such as child soldiering (Fritz et al., 2021; Weierstall & Elbert, 2011).

This gap is particularly notable considering the substantial evidence linking ACEs to dissocial behaviors, delinquency, and violent offending (Craig et al., 2017; Fox et al., 2015; Hilton et al., 2019), with the frequency and multiplicity of ACEs appearing to influence offending trajectories in juvenile offenders, which may contribute to psychopathy-related dispositions (Baglivio et al., 2015, 2020). Such a linkage is further exemplified by the high prevalence rates of ACEs among forensic psychiatric populations. Recent studies have shown that up to 70% of forensic psychiatric patients report a history of ACEs, with specific experiences, such as sexual and physical abuse, reported in 20% and 57% of cases, respectively (Fritz et al., 2021; Laporte et al., 2023). These research findings highlight the complex backgrounds of forensic psychiatric patients, which contribute to the development of psychopathology and violent behavior. Therefore, this study focuses on these factors, with a particular emphasis on appetitive aggression—the enjoyment of violence—which is especially relevant to this population.

In terms of scientific theories, such a contribution has been posited by the attenuation hypothesis (Susman, 2006), which stresses a potential intersection of the psychobiological impact of ACEs and the development of dissocial personality traits.

From a psychodynamic perspective, ACEs are thought to influence the development of maladaptive personality structures. These structures reflect dysfunctional internalized attachment patterns, impaired self-perception, and inadequate coping mechanisms for managing interpersonal stressors (Luyten & Fonagy, 2022). The operationalized psychodynamic diagnosis (OPD) system provides a comprehensive framework for assessing such personality dynamics across five axes, as follows: illness experience and treatment preconditions (Axis I), maladaptive interaction patterns (Axis II), motivational conflicts (Axis III), structural vulnerabilities (Axis IV), and symptom diagnostics (Axis V) (Arbeitskreis zur Operationalisierung Psychodynamischer Diagnostik, 2008).

Axis IV of the OPD system has been adapted for self-report assessment through the OPD-Structure Questionnaire (OPD-SF), which measures cognitive and emotional capacities, attachment capabilities, and self-regulation (Ehrenthal et al., 2012). However, no studies have systematically examined how OPD-based personality structure assessments in subjects who experienced ACEs correlate with specific aggression subtypes. Exploring these bivariate associations could inspire future research to determine whether specific psychological constructs truly account for these connections.

Consequently, this study aims to address the above-listed critical gaps in the literature by investigating the association between ACE-induced, OPD-assessed personality structure deficits and aggression subtypes. Specifically, we hypothesize that individuals with ACEs will exhibit higher OPD-SF scores, reflecting greater personality fragmentation. This fragmentation is expected to predict higher levels of both reactive and appetitive aggression.

## 2. Methods

### 2.1. Sample

In this study, a total of 53 male offenders were surveyed, all of whom were housed between 2019 and 2022 under §64 of the German Criminal Code at the Clinic for Psychiatry and Psychotherapy at the District Hospital (BKH) Günzburg, Bavaria, Germany. Placement under §64 of the German Criminal Code focuses on a two-year, multimodal treatment of convicted offenders, with the goal of resocialization and stabilization of existing substance use disorders to reduce individual criminal recidivism. Such placement is ordered when a court determines that the index offense is directly linked to the substance use disorder. Inclusion criteria were a primary substance use disorder diagnosis, sufficient German language skills, and an age minimum of 18 years. Specific exclusion criteria comprised of intellectual disabilities, organic personality disorders, schizophrenic spectrum disorders, and a lack of language comprehension. Patients did not receive a compensation for participating in the study. As a diagnostic manual, the International Classification of Diseases in its tenth version (ICD-10) was used. All relevant sociodemographic and forensic–psychiatric characteristics can be found in Table 1.

### 2.2. Questionnaires

#### 2.2.1. The OPD Structure Questionnaire (OPD-SF)

The operationalized psychodynamic diagnosis (OPD) assesses various aspects of mental disorders, resources, vulnerabilities, interaction patterns, and personality across five axes. The first axis examines the patient’s subjective experience of illness and the preconditions for treatment. The second axis identifies central maladaptive interaction patterns. The third axis focuses on biographically significant and currently influential conflict and motivational patterns. The fourth axis describes structural vulnerabilities in personality, particularly concerning fundamental psychological and interpersonal skills, as well as processing capacities. The fifth axis incorporates symptom diagnostics based on international classification systems.

The OPD Structure Questionnaire (Ehrenthal et al., 2012) itself consists of 95 items rated on a 5-point Likert scale ranging from 0 (does not apply at all) to 4 (fully applies) and focuses on the fourth axis, which represents the biographically acquired “toolkit” for perception, regulation, communication, and attachment in relation to one’s inner world and other people through self-report. Within this axis, the four following structural dimensions are distinguished: cognitive abilities, encompassing self-perception and object perception; emotional abilities, referring to emotional communication both internally and externally; regulatory capacity, which includes self-regulation and the regulation of object relations; and attachment capacity, addressing both internal and external aspects of attachment.

All subscales demonstrate satisfactory to good reliability, with Cronbach’s alpha ranging from 0.72 to 0.91 (Ehrenthal et al., 2012).

#### 2.2.2. Appetitive and Facilitative Aggression Scale (AFAS)

The AFAS questionnaire measures the constructs of appetitive and reactive aggression, each with 15 items. The original English version of the questionnaire, developed by (Weierstall & Elbert, 2011), includes 15 items specifically targeting appetitive aggression. For the German version, the authors expanded the questionnaire by adding 15 items designed to measure reactive aggression.

The questionnaire retrospectively assesses how frequently participants have exhibited specific aggressive behaviors, thoughts, or feelings throughout their lives. Reactive aggression is measured using items such as “Did you lose control and act out because someone provoked you?”, while appetitive aggression is assessed through items like “Did you provoke others simply because you found it enjoyable?”.

Each item is rated on a 5-point Likert scale (never, rarely, sometimes, often, and very often). Scores for both scales are summed, resulting in a total score for appetitive and reactive aggression, ranging from 0 to 60. Higher total scores indicate higher levels of aggression, while lower scores indicate lower levels.

The reliability of the AFAS has been evaluated as satisfactory to good in the study by Augsburger et al. (2015), with Cronbach’s alpha reported as 0.75 for the reactive aggression scale and 0.85 for the appetitive aggression scale. Furthermore, the questionnaire demonstrates acceptable convergent and divergent validity (Weierstall & Elbert, 2011).

#### 2.2.3. Maltreatment and Abuse Chronology of Exposure Scale–German Version (KERF)

ACEs were assessed using the German version (Isele et al., 2014) of the Maltreatment and Abuse Chronology of Exposure Scale—MACE, (Schauer et al., 2011; Teicher & Parigger, 2015). The KERF is a retrospective self-report questionnaire consisting of 75 items assigned to 10 subscales, which include parental verbal abuse, parental, nonverbal emotional abuse, sexual abuse, emotional neglect, physical neglect, witnessed physical violence toward parents, witnessed violence toward siblings, peer emotional violence, peer physical violence, witnessing violence against siblings, and witnessing violence against a parent.

Each item (for example, “Did your parents lock you in a closet, a storage room, a basement, a garage, or another space, possibly very small and dark?”) is answered with “yes” or “no”. A response of “yes” is coded as 1, and “no” is coded as 0. The total score for each subscale is obtained by summing these values. The authors provide specific cutoff values for each subscale (Isele et al., 2014). In the present study, participants were classified as having experienced maltreatment if their total score in at least one subscale exceeded the cutoff value (parental verbal abuse: 3 of 4 items, parental nonverbal emotional abuse: 3 of 5 items, sexual abuse: 2 of 8 items, emotional neglect: 5 of 10 items, physical neglect: 3 of 5 items, witnessed physical violence toward parents: 2 of 4 items, witnessed violence toward siblings: 2 of 4 items, peer emotional violence: 4 of 5 items, and peer physical violence: 2 of 5 items).

Additionally, respondents are asked to specify the time period during which each adverse childhood experience occurred by selecting an age range between 1 and 18 years. This allows for detailed documentation of the timing (e.g., early or late childhood) and duration of the experiences.

The KERF scale has been validated against the Childhood Trauma Questionnaire (Bernstein et al., 2003), demonstrating strong correlations with the overall scores and subscale scores. However, the authors provide no information on the reliability of the KERF scale.

### 2.3. Ethical Approval

Positive ethics approval has been obtained from the Ethics Committee of the University of Ulm (Application No. 288/17). All participants received oral and written information about the study and provided written informed consent prior to their voluntary participation.

### 2.4. Statistical Analysis

Data were analyzed using IBM SPSS Statistics for Windows, Version 28 (Armonk, NY, USA: IBM Corp.). To determine an appropriate sample size, the study by Ehrental and colleagues (Ehrenthal et al., 2012) was referenced. The authors report the following effect sizes according to Cohen for the comparison of non-clinical and clinical samples: *d*(non-clinical vs. outpatient) = 0.82; *d*(non-clinical vs. inpatient) = 1.50. Using the G*POWER 3.1 program by Buchner, Erdfelder, Faul, and Lang (Faul et al., 2009), a sample size of *n* = 42 (*n* = 21 per group) was determined for a medium effect size of 1.16, a two-tailed test, and a statistical power of 0.95 (1 − β).

First, descriptive statistics (mean, standard deviation, and absolute and relative frequencies) were calculated for all variables. Then, the internal reliability of all questionnaires and scales was assessed for the current sample using Cronbach’s alpha. All scales and subscales were tested for normal distribution. In cases of non-normal distribution, the Mann–Whitney U-test or Spearman correlation was used instead of the *t*-test or Pearson correlation. For the *t*-test for independent samples, the Welch test was applied when the variances in the two groups were unequal, as determined by Levene’s test.

Next, we compared the eight scale values and the total score of the OPD Structure Questionnaire and the two subscales of the AFAS questionnaire between the group of patients with ACEs and those without such experiences.

To analyze which personality components are associated with aggressive behavior, correlations were calculated between the eight subscales and total scale of the OPD Structure Questionnaire and the scales for appetitive and reactive aggression from the AFAS questionnaire. The significance level was set at 5%. To correct for multiple testing, we followed the method described by Benjamini and Hochberg (1995).

## 3. Results

Table 2 provides information on the number of participants with ACEs, the mean age at first trauma, and the duration of these experiences. In the current study sample, 39 partakers (74%) reported at least one adverse childhood experience above the cut-off value for the individual subscales (i.e., total score). The mean duration of ACEs across all subscales was 13.62 years (*SD* = 5.14), and the mean age at first trauma was 3.76 years (*SD* = 3.52).

The mean values and Cronbach’s alpha for the complete participants sample in the scales of aggressive behavior and the OPD personality structure can be found in Table 3.

In the next step, participants were divided into two groups based on the established cut-off values for ACEs. Test subjects with ACEs exhibited significant differences compared to those without such experiences in the context of OPD-SF scales (Table 4). Specifically, the ACE group scored significantly higher on the scales external emotional communication, internal attachment, and external attachment.

Subscale analyses provided deeper insights into these differences. Patients with ACEs demonstrated significant difficulties in expressing their emotions to others (external emotional communication: affect communication, *t*(51) = −2.214, *p* = 0.031) and exhibited a lack of trust in others (internal attachment: internalization, *t*(51) = −3.294, *p* = 0.002). Furthermore, they showed poor selfcare and tendencies toward self-neglect (internal attachment: use of introjects, *t*(51) = −3.046, *p* = 0.004).

While patients with ACEs were also less inclined to accept or seek help, the subscales accepting help (*t*(51) = −1.901, *p* = 0.063) and detaching (*t*(51) = −1.164, *p* = 0.250), under external attachment, did not reach statistical significance when analyzed separately. These findings highlight the challenges in emotional communication and attachment patterns among individuals with ACEs, contributing to a fragmented personality structure. After controlling for Type I error, according to Benjamini and Hochberg (1995), only the attachment inward scale achieves statistical significance.

To address the research question regarding whether individuals with more pronounced personality disintegration are more likely to exhibit reactive or appetitive aggression, Table 5 presents correlations between AFAS subscales and OPD-SF scales. Subjects with higher reactive aggression tendencies demonstrate elevated scores in the self-perception, object perception, self-regulation, the regulation of object relations, inward attachment, outward attachment, and the total OPD-SF score. Conversely, participants with increased appetitive aggression tendencies show higher scores in these same scales, excluding object perception and inward attachment.

## 4. Discussion

In summary, the current research reveals that individuals who have experienced ACEs exhibit significant deficits in many binding-related variables of the OPD-SF. Specifically, they face challenges in expressing emotions, trusting others, engaging in effective interpersonal communication, and maintaining selfcare. Furthermore, they tend to neglect themselves and are less likely to seek help.

Notably, the severity of these deficits—indicative of greater personality disintegration—was strongly associated with higher levels of self-reported aggression. Surprisingly, this relationship held true for both reactive and appetitive aggression. However, differences emerged at the subscale level: object perception and inward attachment were linked to reactive aggression but showed no correlation with appetitive aggression.

The observed connection between deficits across various interpersonal binding aspects and ACEs aligns well with findings by Poole et al. (2018), who reported that ACEs predict emotional dysregulation and interpersonal difficulties in adulthood. Trust issues among individuals with ACEs are similarly supported by the current literature, such as Neil et al. (2022), who identified a deficit in perceiving unfamiliar faces as trustworthy.

Interestingly, the notion of selfcare deficits among individuals with ACEs appears relatively novel. A recent latent class analysis conducted among African American social work students highlighted this issue, revealing negatively heightened stress perception and significantly diminished selfcare behaviors among those with ACEs (Lee et al., 2024). Additionally, a newly published study protocol outlines the development of a FACE self-help app aimed at promoting wellbeing and selfcare in young adults who have experienced ACEs (Brodbeck et al., 2024).

Even more compelling is the finding that ACEs are associated with a weakened self-concept. This is particularly notable given that an intact self-concept functions as a mediator for depression in young adults with ACEs (Hu et al., 2024).

A positive correlation between ACEs and both reactive and instrumental aggression has been consistently reported (Hoeven et al., 2024; Luijkx et al., 2024; Stoppelbein et al., 2024). Interestingly, no such correlation was observed in the current study. The most probable explanation for this is the relatively small sample size, as the increase in AFAS sum scores among participants who experienced ACEs, while evident, did not reach statistical significance.

The cycle of violence theory (Dodge et al., 1990) provides a theoretical framework to explain this phenomenon, positing that insecure attachment patterns, coupled with underdeveloped reasoning abilities, heightened need for appraisal, and impaired attentional control, increase the likelihood of aggressive behavior. Additionally, Bandura’s seminal work on social learning theory (Bandura, 1978) underscores the role of observational learning in linking ACEs to reactive aggression.

In the context of appetitive aggression, the present study provides the first evidence that ACEs—beyond extreme events, such as child soldier experiences (Augsburger et al., 2015; Hecker et al., 2012)—can foster pleasure derived from aggression. At the subscale level, a particularly notable finding is the relationship between difficulties in regulating object relations (from a psychodynamic perspective, early-life relational experiences) and the enjoyment of aggressive behavior.

Another noteworthy observation is the OPD-SF-based distinction between reactive and appetitive aggression, which appears partially influenced by an individual’s capacity to differentiate between inner and outer realities, as well as their ability to perceive others as whole individuals with autonomous rights. Reactive aggression may be driven by the irrational belief that, if one perceives a situation as aggressive, the counterpart shares this perception, thereby justifying violent responses. In contrast, appetitive aggression seems to involve an intact recognition of others as autonomous individuals; however, the perpetrator consciously crosses the moral boundary despite this awareness.

### Limitations

One major limitation of this study is the relatively small sample size of 53 participants, which necessitates viewing the results as preliminary, since such a sample size may not be statistically sufficient to detect small- or medium-sized effects. This should be considered when interpreting the results. In light of this limitation, non-significant results—such as the absence of an association between inward and outward emotional communication and reactive and appetitive aggression—may be due to insufficient statistical power and should be further re-examined in future studies with a larger test population.

Another significant constraint is the study’s correlative design. Given the multitude of factors influencing aggressive behavior (i.e., additional social or genetic variables, as considered in the bio-psycho-social model), drawing causal conclusions focusing exclusively on personality aspects remains challenging. Consequently, even if therapeutic interventions are derived from these findings, their capacity to effect substantial behavioral changes in patients may be limited.

Additionally, self-reported data are susceptible to biases such as socially desirable responses or hidden motives, including deliberate symptom exaggeration or dissimulation (Moller, 2014). However, a meta-analysis by Ray et al. (2013) provides some reassurance, finding no significant response style biases among individuals with high psychopathic traits, who are often associated with manipulative behavior.

## 5. Conclusions

In conclusion, these findings highlight novel therapeutic targets, such as the regulation of object relations and the enhancement of object perception, for psychotherapeutic interventions and aggression prevention strategies.

## Figures and Tables

**Table 1:** Patients’ sociodemographic and forensic–psychiatric characteristics.

Sociodemographic Variables	Patient Sample (*N* = 53)
	*M* (*SD*)/*n* (%)
Age (years)	34.96 (9.10)
**Highest level of education**	
Graduation	6 (11%)
No graduation	32 (60%)
Graduation after 9 years	14 (26%)
Graduation after 10 years	1 (2%)
**Marital status**	
Married/committed partnership	10 (19%)
Short/unstable relationships (<6 months)	2 (4%)
Single	34 (64%)
Divorced	7 (13%)
**Primary diagnosis (ICD-10)**	
F10: Alcohol-related disorders	12 (23%)
F11: Opioid-related disorders	3 (6%)
F12: Cannabinoid-related disorders	4 (8%)
F13: Sedative- and hypnotic-related disorders	1 (2%)
F14: Cocaine-related disorders	8 (15%)
F15: Other stimulant-related disorders	5 (9%)
F19: Multiple substance use disorders	20 (38%)
**Secondary diagnosis of a personality disorder (ICD-10)**	
F60.0: Paranoid	1 (2%)
F60.2: Dissocial	4 (8%)
F60.3: Emotionally unstable	2 (4%)
Age at first inpatient treatment ^1^ (years)	28.83 (10.29)
Number of previous inpatient psychiatric admissions ^2^	1.20 (3.23)
Duration of current detention (in months)	11.99 (8.32)
**Most serious index delict**	
Murder/manslaughter	1 (2%)
Robbery	4 (8%)
Assault	17 (32%)
Fraud/theft	6 (11%)
Drug offences	23 (43%)
Age at first conviction ^3^ (in years)	23.13 (8.35)
Number of previous convictions ^2^	6.35 (6.19)

^1^ missing values = 5, ^2^ missing values = 2, ^3^ missing values = 1; StGB = German Criminal Code, *N/n* = absolute number, *M* = mean, *SD* = standard deviation, % = frequency in percent, ICD-10 = International Statistical Classification of Diseases 10th Version.

**Table 2:** Descriptive statistics of the questionnaire on ACEs (KERF).

Type of Adverse Experience	Above Cut-Off	Age at First Trauma	Duration of Trauma
	*n* (%)	*M* (*SD*)	*M* (*SD*)
Physical abuse by parents	26 (49)	6.30 (3.03)	7.95 (4.41)
Verbal abuse by parents	27 (51)	7.27 (4.67)	9.32 (5.39)
Nonverbal emotional abuse by parents	20 (38)	6.75 (4.40)	8.50 (5.93)
Sexual abuse	9 (17)	9.11 (3.62)	4.33 (5.20)
Emotional neglect	14 (26)	4.18 (5.51)	14.67 (3.32)
Physical neglect	3 (6)	3.67 (4.62)	12.00 (5.57)
Witnessed physical violence between parents	16 (30)	5.00 (3.04)	9.57 (6.15)
Witnessed violence against siblings	21 (40)	7.20 (3.53)	7.40 (4.63)
Emotional abuse by peers	21 (40)	8.19 (4.07)	7.94 (5.21)
Physical abuse by peers	23 (43)	10.10 (4.01)	5.30 (3.42)

*M* = mean, *SD* = standard deviation, *n* = absolute number, % = frequency in percent.

**Table 3:** Descriptive statistics and Cronbach’s alpha for the questionnaires on aggressive behavior (AFAS) and personality structure (OPD-SF).

	*M* (*SD*)	Cronbach’s Alpha
**Aggressive Behavior (AFAS)**		
Reactive aggression	15.82 (10.78)	0.920
Appetitive aggression	11.50 (9.84)	0.900
**Personality Structure (OPD-SF)**		
Self-perception	0.99 (0.60)	0.845
Object perception	1.56 (0.48)	0.815
Self-regulation	1.37 (1.52)	0.811
Regulation of object relations	1.52 (0.63)	0.856
Emotional communication inward	1.36 (0.44)	0.619
Emotional communication outward	1.59 (0.54)	0.804
Internal attachment	1.62 (0.68)	0.760
External attachment	1.94 (0.64)	0.684
Total score	1.49 (0.46)	0.954

*M* = mean, *SD* = standard deviation, OPD-SF = Operationalized Psychodynamic Diagnosis Structure Questionnaire, AFAS = Appetitive and Facilitative Aggression Scale.

**Table 4:** Differences between patients with and without ACEs in the subscales and total score of the OPD-SF and in the subscales of the AFAS.

Questionnaire Subscales	Patients Without Adverse Childhood Experiences (*n* = 16)	Patients with Adverse Childhood Experiences (*n* = 37)	Statistics
	*M* (*SD*)	*M* (*SD*)	
**Aggressive Behavior (AFAS)**			
Reactive aggression	13.23 (9.78)	16.88 (11.14)	*z* = −0.940, *p* = 0.347
Appetitive aggression	8.54 (7.59)	12.67 (10.47)	*z* = −1.283, *p* = 0.199
**Personality Structure (OPD-SF)**			
Self-perception	0.79 (0.48)	1.08 (0.64)	*t*_(51)_ = −1.624, *p* = 0.110
Object perception	1.39 (0.55)	1.64 (0.43)	*t*_(51)_ = −1.797, *p* = 0.078
Self-regulation	1.69 (2.66)	1.24 (0.56)	*t*_(51)_ = 0.671, *p* = 0.326
Regulation of object relations	1.30 (0.62)	1.61 (0.61)	*t*_(51)_ = −1.686, *p* = 0.098
Emotional communication inward	1.33 (0.48)	1.37 (0.43)	*t*_(51)_ = −0.325, *p* = 0.747
Emotional communication outward	1.34 (0.53)	1.70 (0.51)	***t*_(51)_ = −2.315**, *p* = 0.025
Internal attachment	1.19 (0.55)	1.80 (0.64)	***t*_(51)_ = −3.357**, *p* = 0.001
External attachment	1.65 (0.62)	2.06 (0.61)	***t*_(51)_ = −2.201**, *p* = 0.032
Total score	1.33 (0.51)	1.56 (0.42)	*t*_(51)_ = −1.700, *p* = 0.095

*M* = mean, *SD* = standard deviation, *t* = t-distributed test statistic of the *t*-test, *z* = Mann–Whitney U-test statistics. Significant results are highlighted in Bold.

**Table 5:** Statistical correlations between reactive and appetitive aggression and the personality structure of participants.

Questionniare Subscales	Reactive Aggression	Appetitive Aggression
	*r_sp_*	*r_sp_*
**Personality Structure (OPD-SF)**		
Self-perception	0.467 *	0.384 *
Object perception	0.399 *	0.267
Self-regulation	0.464 *	0.362 *
Regulation of object relations	0.622 *	0.583 *
Emotional communication inward	0.282	0.221
Emotional communication outward	0.253	0.161
Internal attachment	0.367 *	0.247
External attachment	0.283	0.288
Total score	0.504 *	0.415 *

*r_sp_* = Spearman rank correlation coefficient, * *p* < 0.05. Significant results are highlighted in Bold.

## Data Availability

The data presented in this study are available on request from the corresponding author.
